# The Energy Metabolism in *Caenorhabditis elegans* under The Extremely Low-Frequency Electromagnetic Field Exposure

**DOI:** 10.1038/srep08471

**Published:** 2015-02-16

**Authors:** Zhenhua Shi, Hui Yu, Yongyan Sun, Chuanjun Yang, Huiyong Lian, Peng Cai

**Affiliations:** 1 Physical Environment Group, Key Laboratory of Urban Environment and Health, Institute of Urban Environment, Chinese Academy of Sciences, 1799 Jimei Road, Xiamen 361021, P. R. China; 2 University of the Chinese Academy of Sciences, 19 Yuquan Road, Beijing 100049, P. R. China

## Abstract

A literal mountain of documentation generated in the past five decades showing unmistakable health hazards associated with extremely low-frequency electromagnetic fields (ELF-EMFs) exposure. However, the relation between energy mechanism and ELF-EMF exposure is poorly understood. In this study, *Caenorhabditis elegans* was exposed to 50 Hz ELF-EMF at intensities of 0.5, 1, 2, and 3 mT, respectively. Their metabolite variations were analyzed by GC-TOF/MS-based metabolomics. Although minimal metabolic variations and no regular pattern were observed, the contents of energy metabolism-related metabolites such as pyruvic acid, fumaric acid, and L-malic acid were elevated in all the treatments. The expressions of nineteen related genes that encode glycolytic enzymes were analyzed by using quantitative real-time PCR. Only genes encoding GAPDH were significantly upregulated (*P* < 0.01), and this result was further confirmed by western blot analysis. The enzyme activity of GAPDH was increased (*P* < 0.01), whereas the total intracellular ATP level was decreased. While no significant difference in lifespan, hatching rate and reproduction, worms exposed to ELF-EMF exhibited less food consumption compared with that of the control (*P* < 0.01). In conclusion, *C. elegans* exposed to ELF-EMF have enhanced energy metabolism and restricted dietary, which might contribute to the resistance against exogenous ELF-EMF stress.

Magnetic fields have important functions in the origin and evolution of life; animals such as homing pigeons and sea turtles utilize magnetic fields to navigate toward a specific location^1^. However, concerns regarding the harmful effects of extremely low-frequency electromagnetic fields (ELF-EMFs) have increased with the rapid urbanization, industrialization, informatization, and the concomitant electromagnetic complexity and interference in the environment.

Since the first publication of a possible link between ELF-EMF and childhood cancer^2^, numerous studies have investigated the biological effects of ELF-EMFs on humans, and most of these studies found potential harmful effects^3^,^4^. Despite the huge amount of experimental data, the molecular targets of ELF-EMF remain obscure and controversial because of the lack of clear and reproducible effects that can be easily quantified or visualized^5^. Therefore, either ELF-EMF exerts minimal biological effects to trigger major responses in the living body or organisms resist the negative effects of ELF-EMF exposure. Energy metabolism enhancement is a typical adaptive response under hypoxia-induced stress^6^ and heavy metal-induced neurotoxicity^7^,^8^. As another environmental factor, ELF-EMF might also influence energy metabolism.

The free-living nematode *Caenorhabditis elegans* has been used as a model organism to study the influences of environment conditions on human health^9^. Thus, we selected *C. elegans* as a model organism in this study. Previous studies proposed that ELF-EMF exposure affects the reproduction and gene expression of *C. elegans*^5^,^10^,^11^,^12^. However, the effects of ELF-EMF at the metabolic level remain unclear to date. The combination of *C. elegans* and metabolomics is a functional genomics tool that can be used to test the molecular effects of pollution/toxicant exposure^13^, metabolic pathways^14^,^15^,^16^, chemical ecology^17^, and biological variation^18^. In the present study, the effects of ELF-EMF exposure on the metabolites of *C. elegans* were investigated using GC-TOF/MS. Subsequently, food consumption, gene expression, and metabolite concentration in *C. elegans* were analyzed to investigate the relations between ELF-EMF exposure and energy metabolism.

## Results

### Evaluating the effects of ELF-EMF exposure on *C. elegans* at the metabolic level

**Figure 1:**
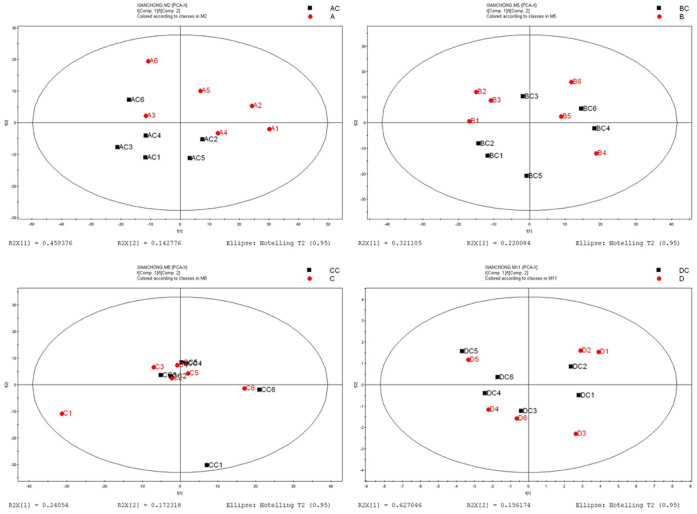
PCA-derived metabolite profile score plots of L4-stage *C. elegans* exposed to ELF-EMF and those of the control. (A) 0.5 mT ELF-EMF exposure versus control. (B) 1 mT ELF-EMF exposure versus control. (C) 2 mT ELF-EMF exposure versus control, and (D) 3 mT ELF-EMF exposure versus control. AC, BC, CC, and DC were the control groups.

**Figure 2:**
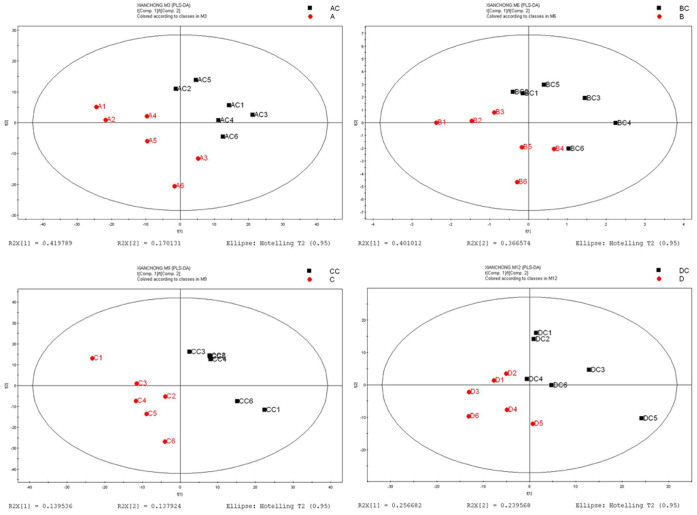
PLS-DA model plots for all groups. (A) 0.5 mT ELF-EMF exposure versus control. (B) 1 mT ELF-EMF exposure versus control. (C) 2 mT ELF-EMF exposure versus control, and (D) 3 mT ELF-EMF exposure versus control. AC, BC, CC, and DC were the control groups.

**Figure 3:**
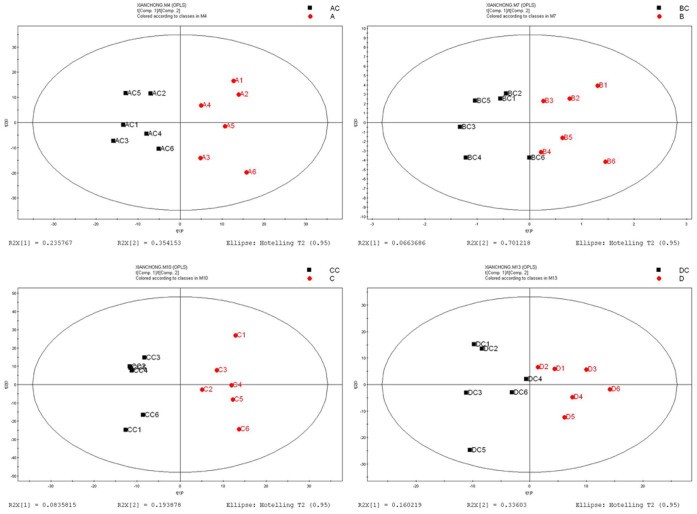
OPLS-DA analysis suggests that the metabolite profile changed in the exposed worms compared with that in the unexposed worms. (A) 0.5 mT ELF-EMF exposure versus control. (B) 1 mT ELF-EMF exposure versus control. (C) 2 mT ELF-EMF exposure versus control, and (D) 3 mT ELF-EMF exposure versus control. AC, BC, CC, and DC were the control groups.

In the metabolomics analysis, six independent pair-wise comparisons were performed to eliminate false positives and negatives, thereby producing robust information on metabolite alteration under ELF-EMF exposure. All data were imported into SIMCA-P+ software (V11.0 Umetrics AB, Umea, Sweden) for processing. As shown in Figure 1, unsupervised principal component analysis (PCA) revealed no noticeable separation between the exposure and control groups. The unit variance-based partial least squares discriminant analysis (PLS-DA) and orthogonal projections to latent structures discriminant analysis (OPLS-DA) as supervised principal component analyses were performed for further analysis. Cross-validation plots for the PLS-DA analyses suggest these models were reliable (Fig. S1). Both PLS-DA and OPLS-DA showed a certain difference between the exposure and control groups (Fig. 2 and 3). These results indicate that the homeostasis of *C. elegans* was disturbed under ELF-EMF exposure, even though the effects were not significant.

As listed in Table 1, the concentrations of metabolites associated with energy metabolism (pyruvic acid, fumaric, and L-malic acids) and neurotransmission (ethanolamine, phenylethylamine, hydroxylamine, and 5-methoxytryptamine) were all increased in all the exposure groups. Moreover, the contents of some amino acids such as alanine, glycine, proline, and leucine were elevated as well. Among all the investigated metabolites, only D-glyceric acid decreased. Both multivariate statistical analysis and metabolite variation analysis showed no regular pattern with increasing magnetic strengths.

### Effects of ELF-EMF exposure on the expression of genes associated with energy metabolism

Nineteen genes encoding enzymes that regulate glycolysis and gluconeogenesis were selected for analysis to check whether or not ELF-EMF enhances energy metabolic rate. As listed in Table 2, only *gpd-1* and *gpd-4* exhibited significant changes in gene expression in worms under ELF-EMF exposure (*P* < 0.01).

**Figure 4:**
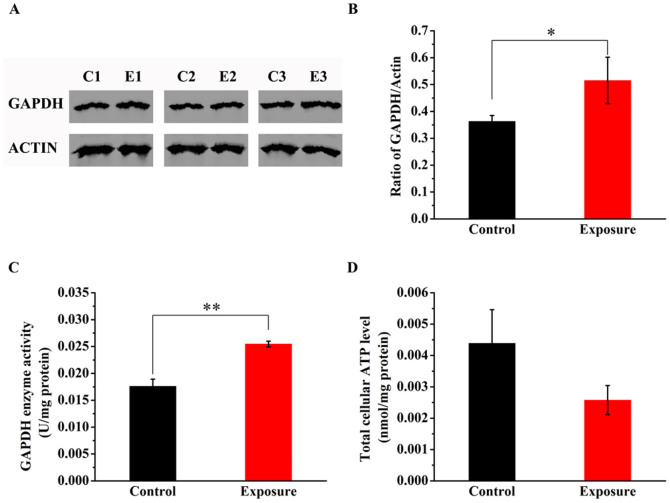
Effects of ELF-EMF exposure on the expression of GAPDH in *C. elegans*. (A) Western blot analysis showing the upregulation of GAPDH protein expression in worms exposed to ELF-EMF compared with the control. (B) Quantitative analysis of panel A showing that the expression of GAPDH is significantly upregulated under ELF-EMF exposure compared with the control (*P* = 0.041). The actin protein was used as an internal control. Values indicate integrated optical density (IOD) ratio of GAPDH/Actin in immunoblots. (C) Enzymatic activity assay for GAPDH. (D) Total cellular ATP level analysis. The exposure group was treated with 50 Hz, 3 mT ELF-EMF. “C” represents “control groups”, while “E” represents “exposure groups”. All data represent three independent experiments. Bars represent SEM of the mean. “*” means *P* < 0.05, compared with the control.

Since the mRNA expression levels of *gpd-1* and *gpd-4* were increased, the endogenous GAPDH present in L4-larva stage worms was examined by western blot, with actin protein as internal control. Our results revealed that GAPDH protein concentration also increased in worms exposed to ELF-EMF compared with control (Fig. 4A, B) (*P* = 0.041).

Other than its indispensable role in energy metabolism, GAPDH is also involved in several non-glycolytic processes^19^,^20^,^21^,^22^. In order to check whether the observed increase in GAPDH protein concentration is to complement the energy metabolism or to achieve any other non-glycolytic functions, intracellular GAPDH enzymatic activity and the total cellular ATP level were tested. In our results, the GAPDH enzymatic activity was increased significantly (*P* < 0.01); however, the total cellular ATP level was lowered approximately 1.5-fold in worms growing under ELF-EMF condition, even though this alteration showed no significance in statistics (Fig. 4C, D).

### Effects of ELF-EMF exposure on food consumption and lifespan of *C. elegans*

**Figure 5:**
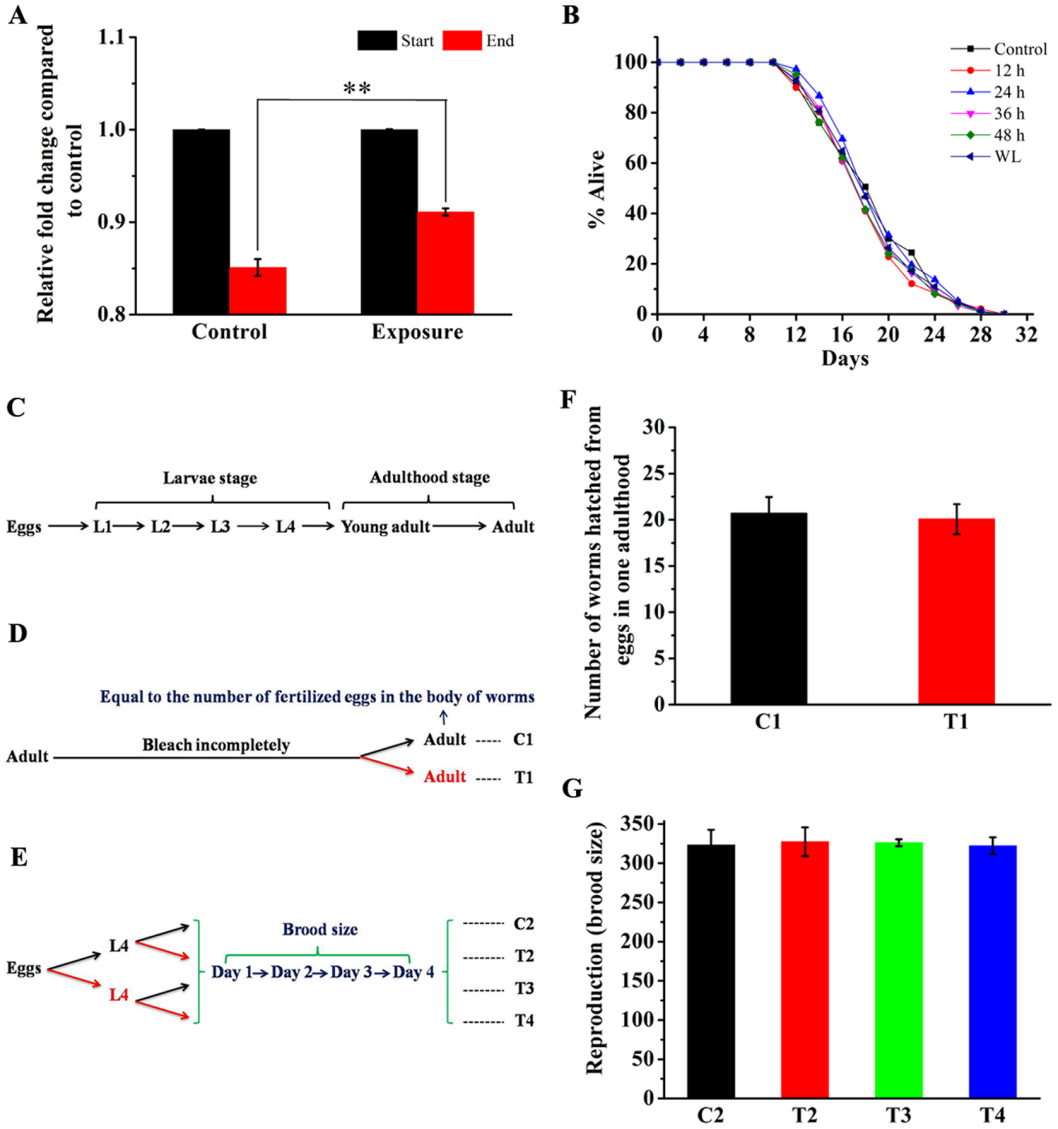
Effects of ELF-EMF exposure on phenotypes. (A) Food consumption analysis. Worms under ELF-EMF exposure have a reduced food intake. (B) Lifespan analysis. Exposed worms at larva stages or whole life have no significant change in lifespan. (C) The life cycle of *C. elegans* consists of the embryonic stage, four larval stages (L1 to L4), and adulthood. (D) Schematic for hatching rate analysis. (E) Schematic for brood size analysis. (F) Hatching rate analysis, ELF-EMF exposure has no effect on hatching rate. (G) Brood size analysis. ELF-EMF exposure has no effect on brood size. The exposure group was treated with 50 Hz, 3 mT ELF-EMF. Bars represent SEM of the mean. “**” represents *P* < 0.01, with respect to the control. “C” represents “control groups”, “T” represents “treatment groups”, “WL” represents “whole life”. Red arrows mean “ELF-EMF exposure”, and black arrows mean “Control” in panels D and E.

*C. elegans* can respond to a variety of stressors including alcohols, heavy metals, sulfhydryl-reactive compounds, salicylate, and heat, by ceasing pharyngeal pumping^23^. The effect of stressors can therefore be conveniently assayed by monitoring the decrease in the density of the bacterial food in liquid cultures of nematodes. In this study, food consumption analysis was also performed on worms exposed to 50 Hz, 3 mT ELF-EMF. Results showed that the changes in OD_600_ in the exposure groups were less than those of the control groups (*P* < 0.01). This result indicates that food intake was restricted in the worms under 50 Hz, 3 mT ELF-EMF exposure (Fig. 5A).

In addition, the lifespans of *C. elegans* exposed to 50 Hz, 3 mT ELF-EMF at the embryogenesis stage (12 h), larval stages (24, 36 and 48 h), and the whole life (WL) span were investigated. No noticeable changes were detected in all exposure groups (Fig. 5B and Table S2).

### Effects of ELF-EMF exposure on the hatching rate and brood size of *C. elegans*

The development of *C. elegans* from fertilization to hatching is referred to as embryogenesis. Post-embryonic development involves growth through four larval stages (L1 to L4) before the final molt to produce the adult^24^ (Fig. 5C). In the hatching rate analysis, the fertilized eggs in the worm's body were chosen for hatching rate analysis, because they can bear ELF-EMF exposure for a longer time than those that have been laid outside, so as to represent the effects of ELF-EMF exposure more accurately. As shown in Figure 5F, the number of worms hatched from the same number of eggs was nearly equal between C1 and T1. This result indicates that 50 Hz, 3 mT ELF-EMF did not affect the hatching rate of nematode eggs.

As an indicator of reproduction, brood size of worms exposed to ELF-EMF in various development stages was measured. No significant differences in the total progeny number were detected among the four exposure groups (Fig. 4G) in this study. These results suggested that ELF-EMF exposure may not affect the reproduction of *C. elegans*.

## Discussion

Metabolites participate in cellular reactions, connecting different pathways that mediate and perform several cell functions; metabolite profiling shows the changes in biological functions or phenotypes in response to genetic or environmental stimuli^25^,^26^,^27^. With this in mind, GC-TOF/MS-based metabolomics was used in conjunction with multivariate statistics to examine the metabolite alteration induced by ELF-EMF exposure. PLS-DA and OPLS-DA analysis showed a clear separation between the groups (with exposure and without exposure), which indicates that ELF-EMF exposure affected *C. elegans* to a certain extent.

Among the analyzed 596 metabolites, 27 metabolites increased their concentrations in all four exposure groups, while only D-glyceric acid decreased. Their concentration variations showed no regular pattern with increasing magnetic strengths in the four groups. The main reason for these results might be that different magnetic fields have different effects on worms, and induce different response and different self-protection bio-processes in worms, which will probably affect the concentrations of related metabolites in turn^28^; meanwhile, different tissues might also be sensitive to different magnetic strengths. Nevertheless, the elevated concentrations of ethanolamine^29^, phenylethylamine^30^, and 5-methoxytryptamine^31^ in all of the treatments imply an adaptive response of *C. elegans* to ELF-EMF exposure, and also indicate that ELF-EMF can induce neurobiological disorder and act as a stressor for *C. elegans*.

Pyruvic acid, fumaric, and L-malic acids are important intermediates in energy generation; increased concentrations of these intermediates might indicate energy metabolism was enhanced. The enhanced energy metabolism contributes to a typical adaptive response under hypoxia-induced stress^6^ and heavy mental-induced neurotoxicity^7^,^8^. It is an ubiquitous mechanism existed in animals and plants^32^. Therefore, energy metabolism enhancement might be conducive to ELF-EMF-induced stress resistance.

For further confirmation of the enhanced energy metabolism, nineteen genes encoding enzymes that regulate glycolysis and gluconeogenesis were selected for gene expression analysis. However, no noticeable changes were observed in the mRNA expression of genes that encode phosphoglycerate kinase, phosphoglycerate mutase, enolase, and pyruvate kinase, which catalyze reactions that produce ATP. The main reason may be that glycolysis uses 10 enzymatic reactions to convert glucose into pyruvate; however, several genes are predicted to be involved in the glycolytic pathway on the basis of homology^33^. Thus, redundancy might contribute to the lack of a detectable gene expression alteration by qRT-PCR.

In *C. elegans*, the highly conserved enzyme GAPDH, which is predicted to reversibly catalyze the oxidation and phosphorylation of glyceraldehyde-3-phosphate to 1, 3-diphosphoglycerate during glycolysis, was encoded by four homologous genes. GPD-1 and GPD-4 are required for embryogenesis and larva development, while GPD-2 and GPD-3 mainly play a role in adulthood^33^,^34^,^35^,^36^. In the present study, GPD-1 and GPD-4 were chosen for gene expression analysis because the samples we studied were L4 larva stage worms. Both of their gene expressions were upregulated significantly (*P* < 0.01) and data from western blot analysis (Fig. 4A) further confirmed the upregulated expression level of GAPDH (*P* < 0.05). However, there is only a minimal increase observed in the fold of protein increase, which does not reflect the increased mRNA levels of *gpd-1* and *gpd-4*. One explanation may be that *gpd-2* and *gpd-3* still have background expressions, which obscured the effect of elevated *gpd-1* and *gpd-4* transcription on GAPDH protein expression level. In addition, the regulation mechanisms during translation and modification process might also resulted in disproportionate variation between *gpd-1* and *gpd-4* mRNA and GAPDH protein expression level.

Theoretically, increased GAPDH expression indicates increased ratios of 1, 3-bisphosphoglycerate to glyceraldehyde-3-phosphate^33^. Glyceraldehyde-3-phosphate accumulation can glycate proteins, leading to deleterious effects within cells^37^. The concentration of glyceraldehyde-3-phosphate available to glycate proteins was lower in the exposure groups than in the control groups. The lower level of such altered protein benefitted the worms exposed to ELF-EMF.

Given that the mRNA expression of most genes involved in glycolysis did not show a significant alteration, we wondered whether the observed increase in GAPDH level in the current study is to complement the energy metabolism or to achieve any other non-glycolytic functions. The elevated enzyme activity indicates that the increased GAPDH expression is probably utilized for complementing the energy metabolism because enzymatic function of GAPDH is contributed to its role in metabolism rather than non-metabolic functions. Moreover, the higher concentration of pyruvic acid also implies enhanced glycolysis, for pyruvic acid is a main product of glycolysis pathway. Taken together, the increased gene/protein expression and the elevated enzyme activity of GAPDH promoted glycolysis pathway in worms exposed to ELF-EMF.

Interestingly, the intracellular ATP level decreased in worms under ELF-EMF exposure, even though the concentration of intermediates involved in glycolysis pathway and TCA cycle were elevated. Given that the food intake was reduced in worms exposed to ELF-EMF, we speculated that GAPDH activity is increased to compensate for the depletion of ATP (due to stress tolerance) but it is still not enough to counteract the effect of diminished food intake and elevated ATP consumption in stress response process. Previous study demonstrated that reduced ATP level was related with increased stress tolerance against high temperature, starvation, or mitochondrial toxicity^38^. Thus, the change of ATP intracellular amount implies a response to the stress caused by ELF-EMF exposure.

Both metabolomics analysis and gene expression analysis showed that the rate of energy metabolism was enhanced in the worms exposed to ELF-EMF. To maintain a balance between ATP demand and supply in energy metabolism with reduced level of substrate, various pathways should be activated to produce energy. To date, numerous works have reported that dietary restriction (DR) nematodes increased their oxygen consumption^39^, and that decreased available nutrients activated nutrient-sensing pathways^40^. A switch in fuel utilization from carbohydrates to short chain fatty acids^41^ and/or a more efficient utilization of ATP^42^ may be responsible for energy metabolism and fundamentally contributes to DR. Moreover, DR has been associated with elevated protein turnover^43^ and higher mRNA levels of GAPDH^44^,^45^, which are in accordance with our results of metabolomics and gene expression analysis, respectively. DR also exhibits many changes that may contribute to physiological benefits, such as reduced oxidative damage, slowed aging-associated decline in DNA repair, altered contents of hormones and induced metabolic changes^46^,^47^. Thus, DR might also contribute to the adaptive response of *C. elegans* to ELF-EMF exposure.

Although variations were found in the level of molecular biology, no significant changes were detected in lifespan, hatching rate and brood size of *C. elegans* under ELF-EMF exposure. A plausible explanation is that the effects of ELF-EMF on *C. elegans* were too tiny that they were covered by worms' adapting system^48^. Furthermore, the relationship between ATP level and phenotype is complicated and still remains unclear. For example, in some nematode mutations, lifespan has a positive correlation with ATP levels^49^, whereas uncoupled relationship also have been observed in some other *C. elegans* mutants^50^,^51^.

**Figure 6:**
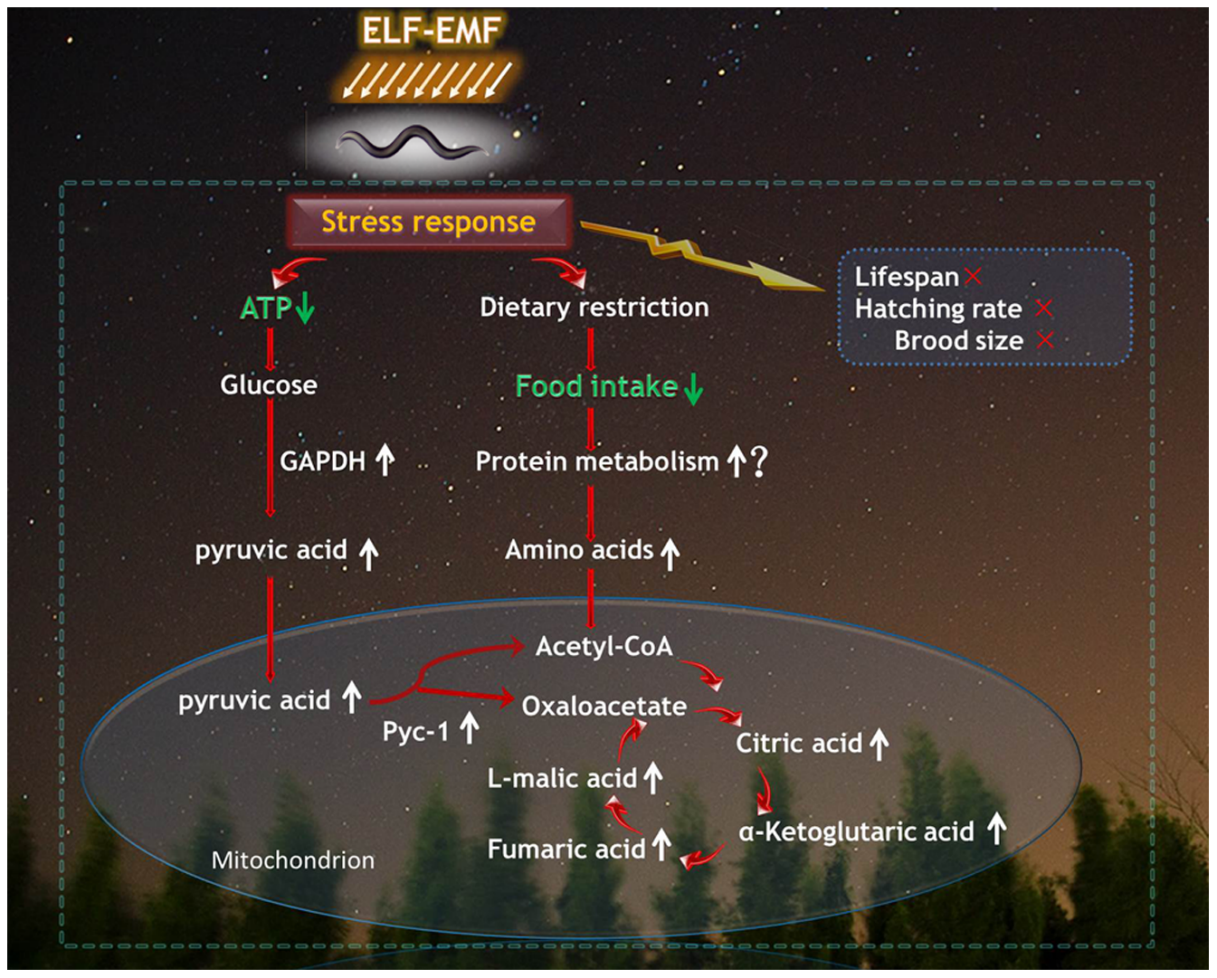
A sketch of our results. Worms under ELF-EMF exposure raise a series of self-protective bio-processes, including reduction of food intake and elevation of ATP consumption, which will consequently lead to the decline of intracellular ATP concentration. Subsequently, the lower level of ATP will promote the process of carbohydrate metabolism (glycolysis and TCA). To meet the requirement of enhanced energy metabolism in the presence of dietary restriction, the protein metabolism might be enhanced, as suggested by the increased concentration of amino acids. “↑” represents “increase”, while “↓” represents “decrease”. “×” means no significant alteration, “?” means a postulation needed further confirmation. Those without arrows were not examined in the present study. The background photograph was sponsored by Mr. San'an Nie.

On the basis of these findings, we propose a biological adaptation model (Fig. 6). Under ELF-EMF stress, self-protective processes expend lots of ATP, and promote the enhancement of energy metabolism, including glycolysis and TCA. DR is also involved in the response to ELF-EMF exposure as an adaptive mechanism. In addition, elevated amino acids concentration suggest that protein metabolism might be enhanced to maintain the concentrations of substrates involved in energy metabolism due to the less food intake in worms under ELF-EMF exposure^41^.

## Conclusion

In this study, we applied metabolomics to consolidate the effects of ELF-EMF on *C. elegans* at the metabolic level, and the elevated concentrations of glycometabolism-related metabolites pyruvic acid, fumaric acid, and L-malic acid indicated that worms under ELF-EMF exposure have an enhanced rate of energy metabolism. We further confirmed this postulation by examining the expression levels of genes that encode enzymes involved in carbohydrate metabolism. Moreover, the reduced food intake demonstrated that DR was involved in the response to ELF-EMF exposure. No significant changes were found in lifespan, hatching rate and reproduction in worms exposed to ELF-EMF, and this might be due to the self-protective mechanisms stated above. As higher organisms including human beings share high homology with *C. elegans* in terms of cellular and molecular structures and functions, metabolic pathways, and developmental processes^52^, humans may also benefit from this mechanism to defend themselves from ELF-EMF stress.

## Methods

### *C. elegans* strains and maintenance

Wild *C. elegans* (N2) was obtained from the Caenorhabditis Genetics Center (University of Minnesota, St. Pau, MN, USA). *C. elegans* strains were cultured on nematode growth media (NGM) seeded with *Escherichia coli* OP50 bacteria. The method for obtaining synchronous cultures was in accordance with standard protocol^53^.

### ELF-EMF exposure system

The ELF-EMF exposure system mainly consists of five parts: power regulator, Helmholtz coil, biochemical incubator, temperature recorder, and condenser. Two vertical coils (150 turns of copper wire measuring 1 mm in diameter) were placed into a biochemical incubator (Jinghong SHP-250, China). The two coils were connected in series, and a 50 Hz sinusoidal magnetic field controlled by a power regulator (PS-7005, China) was generated by feeding a line current. When energized, a uniform magnetic field (0 mT to 3.5 mT) was generated in the center of the coils where the culture dishes were placed. The magnetic flux densities were then measured using a portable field meter (PM 8053B, Italy). A piece of silicone tube connected to a condenser was wound around the coils to counteract the heat that they generated. The temperature was monitored by a thermal resistance probe (sensitivity of ±0.10°C) placed near the culture dishes and recorded every 15 s by a temperature recorder (MIK-214B, China) throughout the entire experiment. In addition, the temperature probes of both biochemical and temperature recorders were positioned to be in contact with the stand holding the Petri dish. The control system comprised the same components as the exposure system, except the former contained no Helmholtz coil.

### Hatching rate and reproduction analysis

Gravid adults were treated with 2% sodium hypochlorite and 0.5 mol/L NaOH to isolate embryos, and then they were transferred into new fresh NGM plates. Upon reaching the maximal egg-laying stage (84 h after synchronization), the worms were bleached incompletely (the worms were dead, and the eggs remained in their bodies) and then transferred into fresh 35 mm NGM plates (Fig. 5D). Each plate had five worms and was randomly distributed into two groups. Each group had at least 10 plates that were randomly divided into two portions. One portion was used as a control (control 1, C1), whereas the other was used for exposure (treatment 1, T1). Finally, the worms were counted after 3 d of culture at 20°C. The processes were repeated at least four times.

To estimate the effects of ELF-EMF exposure on productivity, bleach-prepping gravid adults were transferred into two NGM plates, each with a diameter of 60 mm. One plate was taken for exposure, whereas the other was taken as a control (Fig. 5E). When the eggs reached the L4 larval stage, worms were randomly collected, transferred into fresh 35 mm NGM plates (*n* > 70 and one worm per plate), and then equally divided into two portions. One plate was used as a control (C2 and T3), whereas the other was used for exposure (T2 and T4). The worms that began to lay eggs were transferred to fresh plates daily. The day of the first shift was counted as day 1 in the reproduction assay. After each transfer, the worms that hatched from the eggs were counted to quantify the daily progeny number and total brood size. The process was repeated three times.

### Lifespan analysis

Lifespan tests were performed as previously described by Masse et al.^54^. Worm Lifespan assays were performed at 20°C. Bleach-prepping gravid adults were transferred to six seeded NGM Petri plates. One plate served as a control; the others were exposed to 50 Hz, 3 mT ELF-EMF. One plate was removed from the ELF-EMF exposure system every 12 h up to 48 h. Then, 60 L4-stage worms of each group were selected and transferred to three floxuridine (FUDR)-NGM plates. All plates were placed in a normal biochemical incubator, except for one that was subjected to whole-life (WL) challenge. The day of the shift was counted as day 0 in the adult lifespan assay. A worm was considered dead if it failed to move after being prodded with a platinum wire. The number of dead worms was counted every 2 d until all worms were dead. These experiments were repeated thrice.

### Food consumption analysis

Feeding assays were performed as previously described with minor modifications^55^. *E. coli* OP50 was cultured in LB medium for 12 h. The culture medium was removed by centrifugation and then suspended with M9 buffer (3 g of KH_2_PO_4_, 6 g of Na_2_PO_4_, 5 g of NaCl, 1 mL of 1 M MgSO_4_, H_2_O to 1 L) to OD_600_ at approximately 1.3. Ampicillin was added to inhibit *E. coli* OP50 proliferation. Approximately 1 × 10^3^ pieces of bleach-prepping gravid adults were suspended in 1 mL of M9 buffer and then divided into 10 equal portions. Two portions were transferred to two solid NGM plates for counting the number of eggs, whereas the remaining 8 portions were cultured with 900 μL of *E. coli* OP50 prepared in the last step in two 24 well-plates. One plate was taken for exposure, and another served as a control. The control of each group had the same content except for *C. elegans*, and the total volume was adjusted to 1 mL with M9 buffer. After culturing at 20°C for 72 h, each well was diluted with M9 buffer to the final volume of 5 mL and then mixed uniformly. Aside for 10 minutes for worms deposited at the bottom of Eppendorf tubes, a 200 μL aliquot of the bacterial liquid was pipetted from the top of each Eppendorf tube and then evaluated for OD_600_ using SpectraMax M5 (Molecular Devices, California state, USA). The processes were repeated thrice.

### ATP test

The technique for determining the amount of ATP in the worms used in this study is essentially as described by Le^56^. L4-stage worms (48 h after synchronization) grown under 50 Hz, 3 mT ELF-EMF were harvested (n = 3 × 10^4^) in M9 buffer by centrifugation at 300 g. After the worm pellet was washed with M9 buffer twice, Tissuelyser (NingBo SCIENTZ BIOTECHNOLOGY, CO., LTD, China) was used for 1 min to disrupt tissue. All extraction solvents were centrifuged at 12 000 × g for 5 min at 4°C. The supernatant was transferred to a new 1.5 mL tube for ATP test with the ATP detection kit purchased from Beyotime (Catalog No.: S0026, China). ATP levels were normalized to protein concentration.

### Western blot

Western blot was performed as previously described^57^. In brief, 50-μg of total protein from the L4 stage worms was fractionated by electrophoresis and transferred to polyvinylidene difluoride (PVDF) membranes. The membranes were probed with the mouse-derived anti-GAPDH antibody (60004-1-Ig, Proteintech) and donkey anti-mouse IgG antibody (ab97030, Abcam). Actin was probed with anti-ACTIN antibody (60008-1-Ig, Proteintech) as an internal control. Blots were treated with Western Bright ECL (WBF25, Gel Company, USA) and examined by the chemiluminescence System (Image Station 4000 mm, Kodak, USA). All of the experiments were repeated at least three times.

### GAPDH enzymatic activity assay

GAPDH enzymatic activity was measured as previously described^57^. The supernatant (total protein was 20 μg) containing GAPDH proteins was analyzed for enzymatic activity according to a standard enzyme assay by measuring the change in absorbance at 340 nm that reflects the reaction between NAD+ and NADH. GAPDH enzymatic enzyme activity was tested with the GAPDH enzymatic activity detection kit (SHANGHAI HALING BIOLOGICAL TECHNOLOGY CO., LTD. China).

### RNA extraction and cDNA synthesis

Total RNA was isolated from a synchronized *C. elegans* population using Trizol reagent as previously described with minor modifications^58^. Briefly, L4-stage worms were resuspended in Trizol (1 mL/100 μL compact worms pellet). Protein and lipid impurities were separated from nucleic acids using chloroform, and RNA was precipitated with isopropyl alcohol. The RNA pellet was washed with 75% ethanol, air-dried, and then dissolved in RNase-free water. RNA concentrations were measured using an ND-1000 spectrophotometer (Nanodrop Technology, Wilmington, DE, USA). Total RNA (500 ng) was reverse-transcribed to cDNA following the manufacturer's instructions (cDNA synthesis kit, TaKaRa).

### QRT-PCR measurements

Gene-specific primers were designed using Primer 5.0 software, and *act-1*was selected as the housekeeping gene because its expression was not altered by ELF-EMF treatment. The primers used in this study are shown in Table S1. Quantitative real-time PCR (qRT-PCR) was performed using the SYBR Premix Ex Taq II kit (TaKaRa) and the Roche LightCycle 480 II sequence detection system (Roche, Switzerland). Gene expression studies were performed in triplicate, and the formation of a single PCR product was confirmed using dissociation curves. Negative controls with the primers comprised all components of the PCR mix, except cDNA. Relative fold change in gene expression for each gene was calculated using normalized CT values^58^.

### Metabolite extraction

The methanol–chloroform approach was used for extraction. Geier et al. reported that this approach to results in good overall metabolomic coverage^59^. L4-stage worms (48 h after synchronization) were washed from their culture plates and then harvested by centrifugation at 300 g. *Escherichia coli* OP50 was removed by washing the plates thrice with M9 buffer. Worm pellets were snap frozen by liquid nitrogen and then stored at −80°C. We added 0.4 mL of 3:1 v/v methanol: chloroform to each 2 mL Eppendorf tube containing approximately 4 × 10^4^ of *C. elegans*. QIAGEN Tissuelyser II at 20 Hz was used for 5 min of tissue disruption. All extraction solvents were centrifuged at 10,000 rpm for 10 min at 4°C. The supernatants (0.5 mL) were carefully transferred to 2 mL vials. The supernatants were dried overnight in a vacuum sample concentrator at room temperature. The extraction was derivatized by adding 80 μL of methoxylamine hydrochloride (20 mg/mL) at 37°C for 2.5 h and subsequently adding 100 μL of N, O-bis (trimethylsilyl) trifluoroacetamide at 60°C for 1.5 h. All samples were derivatized in a single batch and then stored at −80°C until analysis.

### GC-TOF/MS analysis

GC-TOF/MS analysis was performed using the Agilent 7890 GC system coupled with Pegasus 4D TOF MS. The system used a DB-5MS capillary column coated with 5% diphenyl cross-linked with 95% dimethylpolysiloxane (30 m × 250 μm inner diameter, 0.25 μm film thickness; J&W Scientific, Folsom, CA, USA). A 1 μm aliquot of the analyte was injected in splitless mode. Helium was used as the carrier gas, and the front inlet purge flow was 3 mL/min. The gas flow rate through the column was 1 mL/min. The initial temperature was maintained at 90°C for 2 min and then increased to 180°C at a rate of 10°C/min, 240°C at a rate of 5°C/min, and 285°C at a rate of 20°C/min for 12 min. The injection, transfer line, and ion source temperatures were 280°C, 270°C, and 220°C, respectively. The energy used was −70 eV in electron impact mode. The MS data were acquired in full-scan mode within the m/z range of 20 to 600 at a rate of 12 spectra/s after a solvent delay of 492 s. The total run time was approximately 34 min per sample. All samples were run in a single batch in the autosampler.

### Metabolite profiling analysis

The total protein mass of animals was used to adjust potential differences in sample size (number of worms and size of worms) between groups. In all the samples, 2-Chloro-L-phenylalanine was loaded as quality control. The data matrix was normalized using an internal control (glutamine) and the total peak area. Data normalization was completed using Excel 2010.

### Data analysis

Statistical analysis was performed using one-way ANOVA. The results of the control and exposure groups were compared using Dunnett's two-sided test (SPSS 16.0, USA). Significance levels were set to *P* < 0.05. In the analysis of metabolite variation, gene expression and food consumption, quantitative normalization within replicates were transformed by the logarithmic base of 2.

## Author Contributions

Conceived and designed the experiments: P.C. and Z.S. Performed the experiments: Z.S., H.Y. and Y.S. Analyzed the data: Z.S. Contributed reagents/materials/analysis tools: P.C., Z.S. and C.Y. Wrote the paper: Z.S., H.L. and P.C. All authors reviewed the manuscript.

## Supplementary Material

Supplementary Information Supplementary information

## Figures and Tables

**Table 1:** Metabolites that have the same change tendency under 0.5, 1, 2, and 3 mT ELF-EMF exposure

	R-concentration			R-concentration			R-concentration			R-concentration		
Metabolites	Control	0.5 mT	Fold changes	Control	1 mT	Fold changes	Control	2 mT	Fold changes	Control	3 mT	Fold changes
pyruvic acid	0.015 ± 0.004	0.023 ± 0.010	0.636	0.018 ± 0.003	0.024 ± 0.008	0.395	0.014 ± 0.006	0.031 ± 0.027	1.148	0.014 ± 0.003	0.020 ± 0.003	0.501*
fumaric acid	0.046 ± 0.005	0.055 ± 0.005	0.274**	0.039 ± 0.008	0.047 ± 0.005	0.242	0.044 ± 0.006	0.051 ± 0.007	0.212	0.044 ± 0.006	0.053 ± 0.004	0.264*
L-malic acid	0.528 ± 0.142	0.615 ± 0.119	0.219	0.503 ± 0.102	0.710 ± 0.107	0.497*	0.488 ± 0.062	0.632 ± 0.150	0.374	0.411 ± 0.120	0.500 ± 0.138	0.283
alanine	1.144 ± 0.474	1.967 ± 0.785	0.782	0.828 ± 0.198	0.960 ± 0.280	0.214	1.022 ± 0.263	2.137 ± 2.107	1.064	0.706 ± 0.191	0.874 ± 0.226	0.308
glycine	0.077 ± 0.042	0.146 ± 0.059	0.911*	0.092 ± 0.042	0.110 ± 0.054	0.263	0.116 ± 0.026	0.202 ± 0.105	0.804	0.065 ± 0.038	0.090 ± 0.047	0.459
leucine	0.006 ± 0.001	0.008 ± 0.001	0.395**	0.005 ± 0.001	0.007 ± 0.002	0.428	0.006 ± 0.001	0.007 ± 0.001	0.214	0.005 ± 0.001	0.006 ± 0.001	0.251*
proline	0.006 ± 0.002	0.007 ± 0.001	0.217	0.005 ± 0.001	0.007 ± 0.001	0.381	0.004 ± 0.001	0.005 ± 0.001	0.308*	0.006 ± 0.001	0.007 ± 0.001	0.383*
3-hydroxy-L-proline	0.039 ± 0.004	0.045 ± 0.005	0.198	0.031 ± 0.010	0.038 ± 0.008	0.320	0.032 ± 0.005	0.035 ± 0.006	0.110	0.026 ± 0.005	0.036 ± 0.008	0.446
ornithine	0.014 ± 0.002	0.016 ± 0.002	0.113	0.013 ± 0.003	0.016 ± 0.002	0.352	0.013 ± 0.002	0.014 ± 0.002	0.074	0.012 ± 0.002	0.016 ± 0.002	0.358
norleucine	0.027 ± 0.003	0.036 ± 0.003	0.398**	0.019 ± 0.007	0.023 ± 0.005	0.265	0.029 ± 0.002	0.034 ± 0.002	0.197*	0.024 ± 0.005	0.029 ± 0.005	0.266
cycloleucine	0.021 ± 0.002	0.025 ± 0.003	0.269*	0.015 ± 0.004	0.017 ± 0.003	0.193	0.021 ± 0.002	0.025 ± 0.003	0.214	0.018 ± 0.002	0.023 ± 0.002	0.339**
malonic acid	0.015 ± 0.005	0.023 ± 0.005	0.621*	0.009 ± 0.003	0.010 ± 0.003	0.131	0.018 ± 0.004	0.026 ± 0.009	0.547	0.013 ± 0.004	0.016 ± 0.004	0.354
dihydrocoumarin	1.077 ± 0.203	1.262 ± 0.237	0.228	0.956 ± 0.221	1.085 ± 0.216	0.182	0.880 ± 0.201	0.972 ± 0.213	0.142	1.033 ± 0.144	1.242 ± 0.233	0.266
spermine	0.054 ± 0.008	0.067 ± 0.007	0.319*	0.035 ± 0.011	0.039 ± 0.010	0.183	0.052 ± 0.005	0.063 ± 0.006	0.271*	0.044 ± 0.006	0.053 ± 0.007	0.292
phenylethylamine	0.045 ± 0.017	0.080 ± 0.030	0.830*	0.036 ± 0.010	0.037 ± 0.010	0.061	0.044 ± 0.008	0.079 ± 0.072	0.832	0.034 ± 0.006	0.040 ± 0.009	0.223
hydroxylamine	1.949 ± 0.848	3.219 ± 1.425	0.724	1.122 ± 0.359	1.354 ± 0.474	0.271	1.881 ± 0.545	4.948 ± 4.811	1.395	1.675 ± 0.404	1.920 ± 0.375	0.197
ethanolamine	0.010 ± 0.007	0.037 ± 0.018	1.843*	0.006 ± 0.001	0.009 ± 0.006	0.683	0.015 ± 0.010	0.026 ± 0.019	0.760	0.008 ± 0.005	0.012 ± 0.007	0.577
2-ketobutyric acid	0.010 ± 0.002	0.013 ± 0.002	0.404*	0.008 ± 0.004	0.012 ± 0.003	0.488	0.009 ± 0.001	0.012 ± 0.004	0.370	0.007 ± 0.002	0.011 ± 0.003	0.715*
creatine	0.026 ± 0.004	0.031 ± 0.002	0.259**	0.015 ± 0.005	0.019 ± 0.004	0.326	0.031 ± 0.005	0.036 ± 0.007	0.195	0.023 ± 0.008	0.032 ± 0.005	0.465
creatine degr	0.032 ± 0.011	0.059 ± 0.015	0.897*	0.022 ± 0.005	0.027 ± 0.008	0.305	0.039 ± 0.009	0.052 ± 0.027	0.421	0.023 ± 0.008	0.030 ± 0.006	0.359
N-methyltryptophan	0.066 ± 0.005	0.075 ± 0.005	0.177*	0.054 ± 0.011	0.069 ± 0.010	0.342	0.057 ± 0.005	0.063 ± 0.011	0.143	0.050 ± 0.006	0.059 ± 0.008	0.233
tetrahydrocorticosterone	0.059 ± 0.008	0.073 ± 0.005	0.309**	0.050 ± 0.015	0.057 ± 0.011	0.194	0.071 ± 0.018	0.078 ± 0.016	0.129	0.047 ± 0.015	0.059 ± 0.014	0.316
myristic acid	0.036 ± 0.014	0.075 ± 0.020	1.055**	0.016 ± 0.007	0.019 ± 0.006	0.274	0.050 ± 0.010	0.074 ± 0.026	0.563	0.032 ± 0.014	0.041 ± 0.012	0.373
fructose	0.038 ± 0.003	0.047 ± 0.004	0.321**	0.025 ± 0.010	0.033 ± 0.009	0.431	0.032 ± 0.004	0.035 ± 0.006	0.149	0.022 ± 0.005	0.031 ± 0.008	0.521
xylose	0.018 ± 0.005	0.022 ± 0.004	0.247	0.015 ± 0.004	0.020 ± 0.004	0.406	0.014 ± 0.002	0.015 ± 0.002	0.157	0.015 ± 0.002	0.019 ± 0.003	0.290
heptadecanoic acid	0.057 ± 0.029	0.133 ± 0.039	1.235**	0.030 ± 0.010	0.042 ± 0.012	0.483	0.103 ± 0.042	0.140 ± 0.062	0.445	0.040 ± 0.024	0.058 ± 0.018	0.562
tetracosane	0.018 ± 0.005	0.027 ± 0.003	0.547**	0.009 ± 0.005	0.012 ± 0.007	0.483	0.022 ± 0.004	0.026 ± 0.002	0.230	0.012 ± 0.004	0.019 ± 0.005	0.600*
D-glyceric acid	0.009 ± 0.003	0.008 ± 0.002	−0.274	0.015 ± 0.005	0.012 ± 0.006	−0.238	0.010 ± 0.001	0.007 ± 0.003	−0.384	0.011 ± 0.003	0.010 ± 0.003	−0.222

The relative concentration of each metabolite is arithmetic mean ± SEM of the data from six biological replicates using GC-TOF/MS. The fold changes were calculated using the formula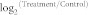. “R” represents “Relative”. * represents *P* < 0.05, ** represents *P* < 0.01.

**Table 2:** Expression level of enzymes related to carbohydrate metabolism

		Relative expression		
Mammalian metabolic enzymes of following	*C. elegans* genes	control	exposure	Sig.
Hexokinase	C50D2.7	1.03 ± 0.13	0.89 ± 0.12	-
Phosphoglucose isomerase	gpi-1	1.00 ± 0.10	0.90 ± 0.08	-
Phosphofructokinase	fbp-1	1.00 ± 0.04	0.99 ± 0.11	-
Aldolase	aldo-1	1.01 ± 0.08	0.95 ± 0.06	-
	aldo-2	1.01 ± 0.12	1.05 ± 0.15	-
Triose-phosphate isomerase	tpi-1	1.01 ± 0.12	0.91 ± 0.15	-
Fructose bisphosphatase	fbp-1	0.99 ± 0.11	0.80 ± 0.12	-
Glyceraldehyde phosphate dehydrogenase	gpd-1	1.00 ± 0.11	2.07 ± 0.63	**
	gpd-4	1.01 ± 0.16	1.96 ± 0.54	**
Phosphoglycerate kinase	pgk-1	0.98 ± 0.06	0.88 ± 0.09	-
Phosphoglycerate mutase	F57B10.3	1.00 ± 0.07	1.00 ± 0.12	-
Enolase	enol-1	1.01 ± 0.17	0.79 ± 0.16	-
Pyruvate kinase	pyk-1	1.00 ± 0.07	1.05 ± 0.08	-
	pyk-2	1.01 ± 0.13	1.03 ± 0.14	-
Pyruvate dehydrogenase A	T05H10.6	1.01 ± 0.22	0.83 ± 0.14	-
Pyruvate dehydrogenase B	C04C3.3	0.97 ± 0.06	0.83 ± 0.09	-
Dihydrolipoamide dehydrogenase	F23B12.5	1.01 ± 0.07	0.88 ± 0.08	-
PEPCK-C	pck-1	1.02 ± 0.20	0.92 ± 0.27	-
Pyruvate carboxylase	pyc-1	1.06 ± 0.19	1.49 ± 0.48	-

The exposure group was treated with 50 Hz, 3 mT ELF-EMF. Value of relative expression is shown in the form of mean fold change ± SEM. ** represents *P* < 0.01, with respect to the control, “-” represents “no significant in statistics”. Each experiment was repeated six times independently.
