# Supplementary material for: The Energy Metabolism in Caenorhabditis elegans under The Extremely Low-Frequency Electromagnetic Field Exposure
Source: Sci Rep. 2015 Feb 16;5:8471. doi: 10.1038/srep08471 (PMC4329544; doi:10.1038/srep08471)
Supplement: Supplementary Information [file srep08471-s1.pdf]

# Supplementary Information

## The Energy Metabolism in *Caenorhabditis elegans* under The Extremely Low-Frequency Electromagnetic Field Exposure

Zhenhua Shi[1,2], Hui Yu[1], Yongyan Sun[1,2], Chuanjun Yang[1], Huiyong Lian[1], Peng Cai[1,*]

[1]Physical Environment Group, Key Laboratory of Urban Environment and Health, Institute of Urban Environment, Chinese Academy of Sciences, 1799 Jimei Road, Xiamen 361021, P. R. China;

[2]University of the Chinese Academy of Sciences, 19 Yuquan Road, Beijing 100049, P. R. China;

[*]To whom correspondence should be addressed. E-mail: pcai@iue.ac.cn

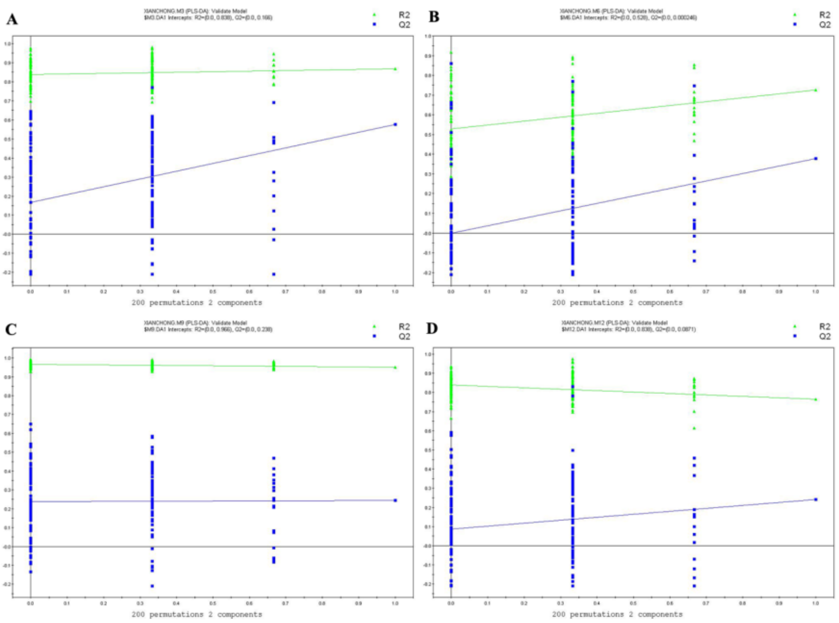


**Figure S1.** Permutation test results for PLS-DA model. (A) For 0.5 mT, (B) For 1 mT, (C) For 2 mT, and (D) For 3 mT. Two hundred Permutations were Performed, and the resulting $R^2$ and $Q^2$ value were Plotted.

**Table S1.** The primers used are listed as follows.

| Genes | Forward primer (5'- 3') | Reverse primer (5'- 3') |
|---|---|---|
| actin-1 | GCTGGACGTGATCTTACTGATTACC | GTAGCAGAGCTTCTCCTTGATGTC |
| C50D2.7 | GTTCTTGTATGCCGTTTA | CTACCAATGAGTGGGTCT |
| gpi-1 | GCACTTTGTCGCACTTTC | CACCAACCCAATCCCAGA |
| pfk-1 | GCAGCCGTCCGCTCTTAT | CTCCGTTCATTGCCCAGT |
| aldo-1 | TACATCTCGGGAGTCATCAT | ACGACACCCTTGTCAACC |
| aldo-2 | GAACTCTTCTCAAGCCAAAC | TTACGGTAGCCAATCCAA |
| tpi-1 | TGCTACAAAGTGCCAAAGG | CATGACGACGTTCCGAGT |
| fbp-1 | GCCTGGAAAGGAAATGGT | CGATGGAAGGGTCAAGAGT |
| gpd-1 | CACCAGATGTTTCCGTCGTT | GGCGAGGATTCCCTTCAT |
| gpd-4 | ATCTTCAAGGAGGAGCCA | GCACGATGCGTTAGAAATA |
| pgk-1 | AGTTCAAATCCATCTCCCAGTG | GGCCCAACATCAAGTCCC |
| ipgm-1 | TGGCAGTCAAGAACAACA | CACCTCCGTCAGAAACAA |
| enol-1 | TTGCCACTGGACAAATCA | CAGCGTAAACAGCATCAG |
| pyk-1 | TTGGGAAGCAGAAAGGGAGTC | TTCGGATGAATGAAGCAAAGAT |
| pyk-2 | ACTATCGGACCAGCGTGTA | GCATGTGCCTCGTGAGTT |
| pdha-1a | ATGAAGCGTTGAAGATTG | ATGGCTGGACGATAGTTT |
| pdha-1b | GAAGAAGCACGGTGATAAG | TGAACTCGCAAATTGGAC |
| dlat-1 | GAAGTGGACCAGGAGGAC | CCAGATACGGCTTGAGTT |
| pck-1 | AGAAGAGTCGCTGGTGGA | ACTTGGGTGTCGAGGAAG |
| pyc-1 | GAACGGTCCAACTACTCCT | TCCTGGTCTACTGCGAAC |

**Table S2.** Results of lifespan.

| Groups | M (day) | m (day) | n | SD |
|---|---|---|---|---|
| Control | 30 | 20.94 | 182 | 2.44 |
| 12 h | 30 | 20.34 | 151 | 1.73 |
| 24 h | 30 | 21.32 | 164 | 1.09 |
| 36 h | 30 | 20.78 | 139 | 2.33 |
| 48 h | 30 | 21.87 | 178 | 2.8 |
| WL | 30 | 20.9 | 174 | 2.62 |

M: maximum life; m: mean life; n: number; SD: standard deviation of mean life; WL: whole life
